# Visualization of heterogeneity and regional grading of gliomas by multiple features using magnetic resonance-based clustered images

**DOI:** 10.1038/srep30344

**Published:** 2016-07-26

**Authors:** Rika Inano, Naoya Oishi, Takeharu Kunieda, Yoshiki Arakawa, Takayuki Kikuchi, Hidenao Fukuyama, Susumu Miyamoto

**Affiliations:** 1 Department of Neurosurgery, Kyoto University Graduate School of Medicine, Kyoto, Japan; 2 Human Brain Research Center, Kyoto University Graduate School of Medicine, Kyoto, Japan; 3 Department of Psychiatry, Kyoto University Graduate School of Medicine, Kyoto, Japan; 4 Center for the Promotion of Interdisciplinary Education and Research, Kyoto University, Kyoto, Japan

## Abstract

Preoperative glioma grading is important for therapeutic strategies and influences prognosis. Intratumoral heterogeneity can cause an underestimation of grading because of the sampling error in biopsies. We developed a voxel-based unsupervised clustering method with multiple magnetic resonance imaging (MRI)-derived features using a self-organizing map followed by K-means. This method produced novel magnetic resonance-based clustered images (MRcIs) that enabled the visualization of glioma grades in 36 patients. The 12-class MRcIs revealed the highest classification performance for the prediction of glioma grading (area under the receiver operating characteristic curve = 0.928; 95% confidential interval = 0.920–0.936). Furthermore, we also created 12-class MRcIs in four new patients using the previous data from the 36 patients as training data and obtained tissue sections of the classes 11 and 12, which were significantly higher in high-grade gliomas (HGGs), and those of classes 4, 5 and 9, which were not significantly different between HGGs and low-grade gliomas (LGGs), according to a MRcI-based navigational system. The tissues of classes 11 and 12 showed features of malignant glioma, whereas those of classes 4, 5 and 9 showed LGGs without anaplastic features. These results suggest that the proposed voxel-based clustering method provides new insights into preoperative regional glioma grading.

Gliomas are the most common primary brain tumor with poor prognosis and are graded according to the classification by the World Health Organization (WHO) of the most malignant region. Tumor grading is important for deciding the treatment, including surgical resection, adjuvant radiation, and chemotherapy. The 5-year survival rate of patients with low-grade gliomas (LGGs) (grade II) is 42–92%^1^, whereas patients with high-grade gliomas (HGGs) (grades III and IV) have a worse prognosis^2^. In particular, glioblastomas (grade IV) develop rapidly^3^, and the 5-year survival rate of patients with these tumors is only 2%^4^. The histologic findings of glioblastomas include diffuse infiltration and simultaneous necrosis in different parts of the tumor. Owing to their heterogeneity, the initial diagnosis by biopsy differs from the diagnosis by total resection in 38% of cases^5^. Previous studies have attempted to grade tumors as a whole; therefore, they could not obtain local information on tumors. If the grade of each region is identified preoperatively, then neurosurgeons can clarify the target for biopsy and the parts of the tumor that need to be resected or preserved, thereby preserving motor or language function.

Magnetic resonance imaging (MRI) is essential for noninvasive diagnosis of the existence, extent, and characteristics of brain tumors. T1-weighted imaging (T1WI), contrast-enhanced T1-weighted imaging (T1WIce), T2-weighted imaging (T2WI), fluid-attenuated inversion recovery (FLAIR), and diffusion-weighted imaging (DWI) sequences are generally performed before surgery. These conventional images can yield a large amount of useful information on tumors, such as tumor morphology, the presence of enhancement, intratumoral hemorrhage, or edema, and are helpful for predicting the tumor grade. It is still controversial whether the presence of enhancement indicates malignancy^6^,^7^,^8^. Contrast enhancement is nonspecific to malignancy and primarily reflects the passage of contrast material across a disrupted blood–brain barrier^9^. Despite this uncertainty, we often examine T1-enhancing regions to determine the target area for the biopsy. Therefore, the most malignant tissue section may not be obtained. The peritumoral hyperintensity on T2WI or FLAIR is also nonspecific, representing tumor infiltration, vasogenic cerebral edema, or both^10^,^11^. These previous studies suggest that glioma grading using only a single feature in MRI is not accurate.

Recently, some studies have applied pattern recognition methods using multiple features to predict tumor grading^12^,^13^. However, these methods demonstrate several problems in clinical application. Pattern recognition methods have two types of clustering, namely supervised and unsupervised, and previous studies have applied either of them. Supervised methods require *a priori* knowledge of boundaries and tissue signatures, whereas unsupervised methods do not^14^. Because methods for preoperative assessment of tumor pathology have not yet been developed, supervised labeling can be inaccurate. Although unsupervised methods are useful and not arbitrary, complicated features, such as those used in previous studies^12^,^13^, make it difficult for clinicians to recognize the most sensitive features for grading. Therefore, unsupervised techniques with multiple and simple features are more useful and suitable for clinical application.

We have recently reported that the multiple features calculated from voxel-based diffusion tensor images (DTI) and clustered by a two-level clustering approach, an unsupervised clustering method with a self-organizing map (SOM)^15^ followed by K-means (KM)^16^, can effectively differentiate between LGGs and HGGs^17^. SOM is a well-known type of neural network unsupervised learning^15^ that simplifies features and shows good visualization of results for data understanding and survey using component planes^18^,^19^. In addition, features that have similar patterns can be identified by KM clustering for the results of SOM. This two-level clustering approach has two important benefits in terms of noise reduction and computational cost. First, because the KM algorithm is very sensitive to outliers^20^, any outliers can adversely affect the accuracy of the clustering. When an SOM is applied prior to KM, outliers can be filtered out, improving the clustering accuracy. Second, the computational time of the two-level clustering approach is considerably shorter than that of KM alone^17^. However, DTI has some limitations such as distortions induced by susceptibility artifacts and low-spatial resolution; hence, the use of DTI in clinical situations or in a navigational system becomes difficult, thereby automatically providing real-time cross-sectional images on conventional MRIs of the brain intraoperatively. To circumvent the problems of DTI, imaging methods that show less distortion and higher spatial resolution, such as T1WI, T1WIce, T2WI, and FLAIR, were used in this study. The higher spatial resolution of these images is suitable for an intraoperative neuronavigational system because the preoperative voxel-based clustered images will have been correctly combined with the preoperative conventional MRI as much as possible to obtain tissue section for each class. Furthermore, the high-spatial resolution leads to the possibility of regional glioma grading. We aimed to develop novel MR-based clustered images (MRcIs) using our two-level clustering approach with multiple features in conventional MRI, such as T1WI, T1WIce, T2WI, and FLAIR, and to use those images to visualize the regional glioma grading because these conventional MRIs are more familiar to neurosurgeons and are easier to understand. We also aimed to determine if our method can correctly predict glioma grading preoperatively in a supervised manner. We demonstrate the possibility of regional glioma grading confirmed by pathological examination with an MRcIs-based neuronavigational system in new patients with glioma.

## Results

### Visualization of unsupervised clustering map

**Figure 1:**
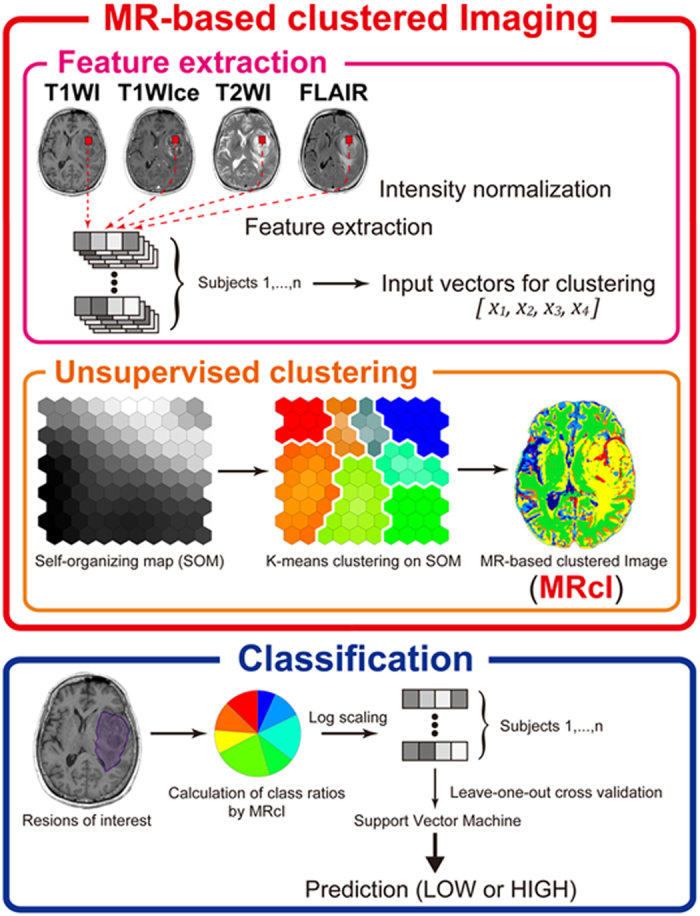
Study procedure for MRI data of glioma.

**Figure 2:**
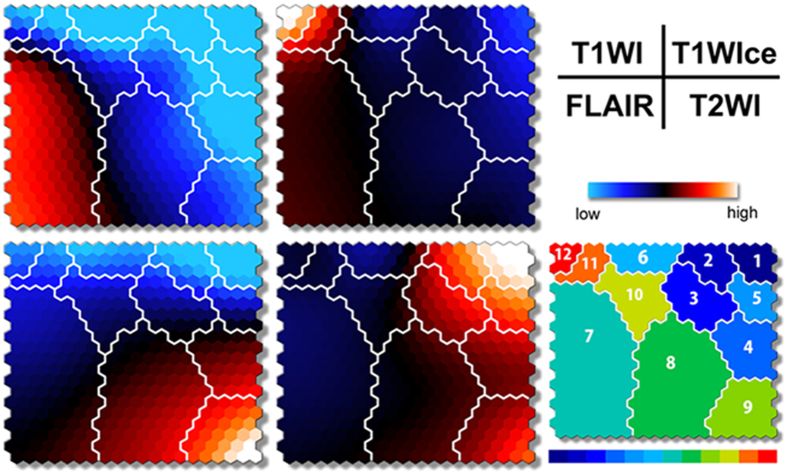
Component planes with SOM ranging from blue to red according to intensities in each MRI value. The inter-class borderlines obtained by KM++ with *K* = 12 are shown on the SOM component planes as white lines between the nodes. Detailed intensity profiles can be seen on the SOM component planes and patterns in each class (from 1 to 12) on the illustrative map (lower right).

We retrospectively reviewed 36 patients, including 21 patients with HGGs and 15 patients with LGGs (Table 1), who underwent T1WI, T1WIce, T2WI, and FLAIR before tumor resection. An overview of the study procedure is depicted in Fig. 1. The component planes of the four MRI variables from T1WI, T1WIce, T2WI, and FLAIR by the SOM analysis show the information of each sequence in each map unit as well as the associations between the clusters and variables^18^ (Fig. 2). The component planes differed from each other. For example, for 12 clusters, T2WI values in the classes 1–5 and 9 were higher than those of the other classes, whereas FLAIR values in the classes 4 and 9 were higher than those of the other classes. T1WIce values in the classes 11 and 12 and T1WI values in class 7 were higher than those of the other classes.

**Figure 3:**
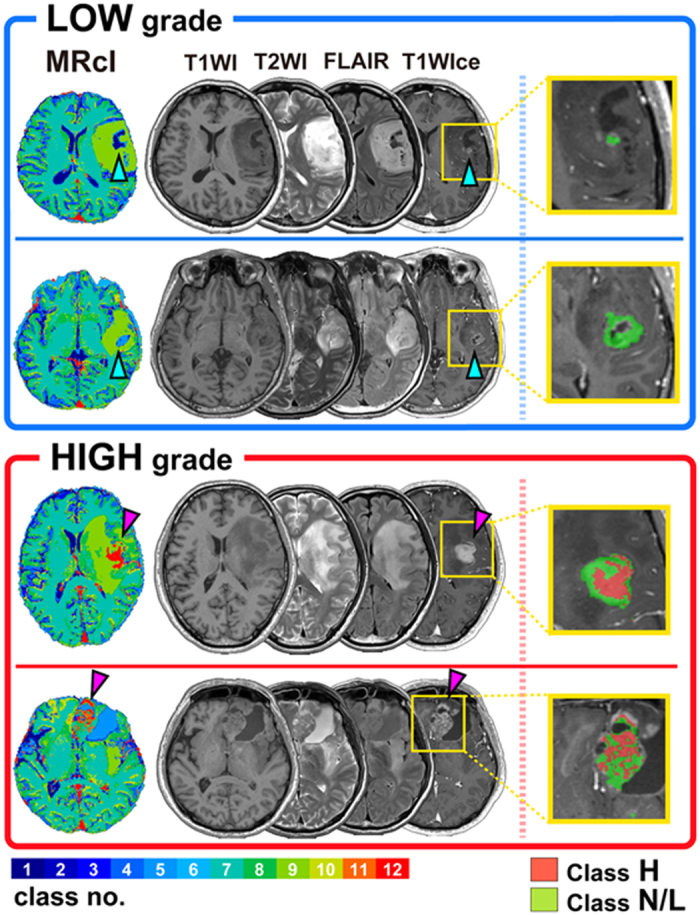
Representative cases of low- and high-grade gliomas, including the 12-class MRcIs, which showed the highest classification performance. The MRcIs, T1WI, T1WIce, T2WI, and FLAIR are shown for each patient. Each color on the MRcIs corresponds to each class in the 12-color bar (left hand corner). Inside the enhanced tumor regions, Classes H and L/N are shown in red and green, respectively, on the enlarged T1WIce (right).

Figure 3 shows representative cases of LGGs and HGGs. Although the voxels of the classes 11 and 12 can be seen in the abnormal areas of LGGs and HGGs, most of them were linearly consecutive, and the raw T1WIce showed that some of them were enhanced vessels. However, in HGGs, voxels of the classes 11 and 12 without vessels were observed, which spread like a stain within the tumor (pink arrow in Fig. 3). Conversely, enhanced regions were not always seen to cluster in the classes 11 or 12, particularly in LGGs (light blue arrow in Fig. 3). Thus, a clear differentiation between LGGs and HGGs could be observed on the MRcIs.

### Classification performance using MRcI

**Figure 4:**
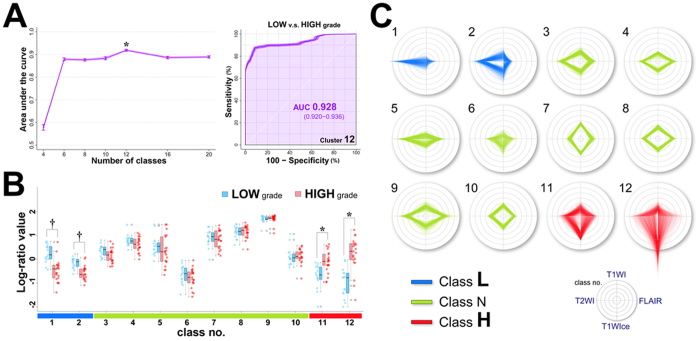
(**A**) Plots of AUC versus the value of *K* in the KM method. The values are presented as the mean values of AUCs. The error bars are 95% CIs (light purple-shaded area). *p < 0.001 (versus all the rest), one-way analysis of variance followed by Tukey’s multiple comparison tests. The 12-class MRcIs significantly showed the highest AUC (left). The ROC curves (dark purple line) in the 12-class MRcIs with AUC and 95% CIs are illustrated by the darker purple-shaded area for classification between low-grade and high-grade gliomas (right). (**B**) Strip chart and box plots showing the median, interquartile range, inner fence, and outliers (circles) for the log-ratio values of each class by 12-class MR-based clustered images of low-grade (blue) and high-grade (red) gliomas. ^†^*p* < 0.001 (Class H < Class L), *p < 0.001 (Class L < Class H), exact Wilcoxon–Mann–Whitney tests. The horizontal color bar on the bottom shows Class L (class 1 and 2) in blue, Class N (class 3–10) in green, and Class H (class 11 and 12) in red. (**C**) Radar charts for MR-based variables in each class by 12-class MRcIs categorized into the following three simplified groups: Classes L, N, and H.

The results of leave-one-out cross-validation (LOOCV) that was used to assess the classification performances using MRcIs and support vector machines (SVM) are shown in Fig. 4A (left). The differences in the areas under the curve (AUCs) were significant among the classes [F(6, 693) = 1147.4; *p* < 10^−16^, 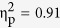]. Tukey’s post-hoc tests showed that the AUC was significantly higher for the 12-class MRcIs than that for other classes (*p* < 0.001). There were no significant differences in AUCs among other classes, except for the 4-class MRcIs. The tests also showed that the AUC was significantly smaller for the 4-class MRcIs than that for other classes (*p* < 0.001). The AUC of the 12-class MRcIs was the largest among the classes [0.928; 95% confidence interval (CI) = 0.920–0.936] (Fig. 4A; right). The sensitivity, specificity, and accuracy of the 12-class MRcIs were 0.81 (95% CI = 0.808–0.812), 0.937 (95% CI = 0.928–0.946), and 0.863 (95% CI = 0.859–0.867), respectively. In contrast, the AUC of the 4-class MRcIs was considerably smaller (0.578; 95% CI = 0.560–0.597). There were no significant group differences in AUCs among the 6-, 8-, 10-, 16-, and 20-class MRcIs (0.882, 0.876, 0.864, 0.887, and 0.888, respectively).

### Differences in the ratio of each class

The log-ratio values of each class of the 12-class MRcIs with the highest classification performances were compared between LGGs and HGGs (Fig. 4B). The values of the classes 1 and 2 were significantly higher in LGGs than in HGGs (*p* < 0.0001, *r* = 0.64; *p* < 0.001, *r* = 0.61; respectively), whereas the values of the classes 11 and 12 were significantly higher in HGGs than in LGGs (*p* < 0.001, *r* = 0.56; *p* < 0.0001, *r* = 0.67; respectively). Furthermore, in the 12-class MRcIs, the values for the classes 11 and 12 were significantly higher in HGGs than in LGGs; similar patterns were observed for the 8- to 16-class MRcIs. Conversely, values of the classes 1 and 2 were significantly higher in LGGs than in HGGs; similar patterns were also observed for the 10- to 20-class MRcIs (Supplementary Fig. S1).

### Differences in the features of each class

**Figure 5:**
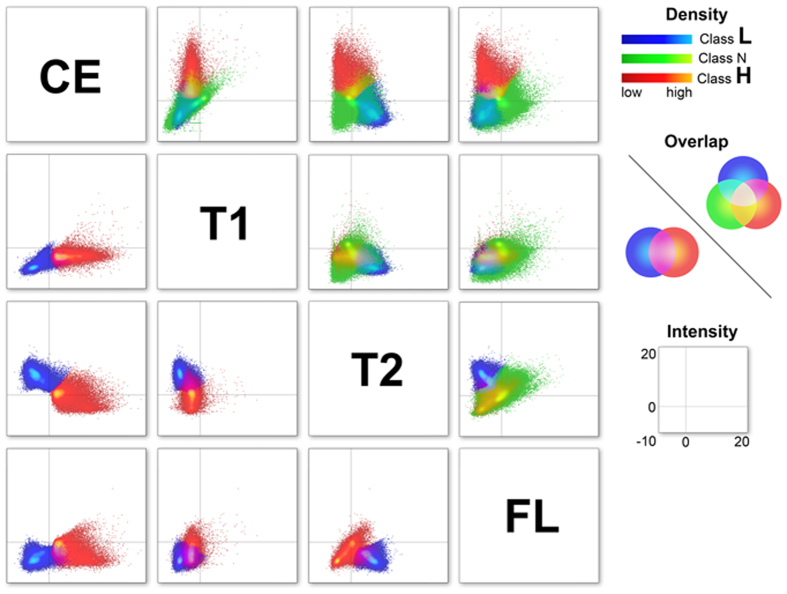
Scatter plot matrix showing the relationships in Classes L, N, and H between the normalized intensity values in T1WIce (CE), T1WI (T1), T2WI (T2), and FLAIR. All three classes are shown in the right upper triangle (blue, green, and red), whereas only Classes H and L are shown in the left lower triangle (blue and red).

We further grouped the 12-class MRcIs into three subclasses to simplify the relationships among them, namely “Class L” (classes 1 and 2), “Class N” (classes 3–10), and “Class H” (classes 11 and 12). Figure 5 shows the relationships in the T1WIce-, T1WI-, T2WI-, and FLAIR-normalized intensity values between the Class L, N, and H using a scatter plot matrix. The distributions of Class H were totally different from those of Class L on the scatter plots of T1WIce/T1WI and T1WIce/T2WI (Fig. 5; lower left triangle). The distributions of Classes L and H for T1WIce/FLAIR, T1WI/T2WI, and T2WI/FLAIR also differed, with partial overlaps. Regarding the three Classes shown in the right upper triangle in Fig. 5, Class N ranged with partial overlaps, particularly for T1WIce/T2WI and T1WIce/FLAIR. Class N widely overlapped with Class L for T1WIce/T1WI and with Class H for T1WI/T2WI, T1WI/FLAIR, and T2WI/FLAIR. Although the relationships among the four MRI features are complicated, we found that grading was dependent on the T1WIce values as well as the relationships among the four features. Furthermore, within the enhanced tumor regions, Class N was found in both LGGs and HGGs, whereas most of the cases of Class H within the tumors were found in HGGs (Fig. 3; right column). The ratios of the normalized intensities of the T1WI and FLAIR images within the enhanced tumor regions were significantly higher in Class H than in Class L (*p* < 10^−16^, *r* = 0.19; *p* < 10^−16^, *r* = 0.21; respectively) (Supplementary Fig. S2). Conversely, the ratios of T2WI intensities within the enhanced tumor regions were significantly higher in Class L than in Class H (*p* < 10^−16^, *r* = 0.18). Even within the enhanced tumors, the Classes L and H showed different patterns for the features, including T1WI, T2WI, and FLAIR. These results also strengthen the importance of the relationships among the four MRI features with regard to grading.

### Individual patterns in the MRI-derived features

The radar charts of the individual normalized intensities of the four MR sequences for each class in the 12-class MRcIs with the highest classification performances are shown in Fig. 4C. The chart patterns of the classes 11 and 12, which were significantly higher log-ratio values in HGGs than in LGGs, comprised high T1WIce values. Conversely, the classes 1 and 2, which were significantly higher in LGGs than in HGGs, had higher T2WI values than T1WI, T1WIce, and FLAIR values. However, although class 5 had a radar chart pattern similar to class 1, there were no significant differences in log-ratio values between LGGs and HGGs in class 5 (*p* = 0.20, *r* = −0.22).

### MRcI-guided tissue sampling

**Figure 6:**
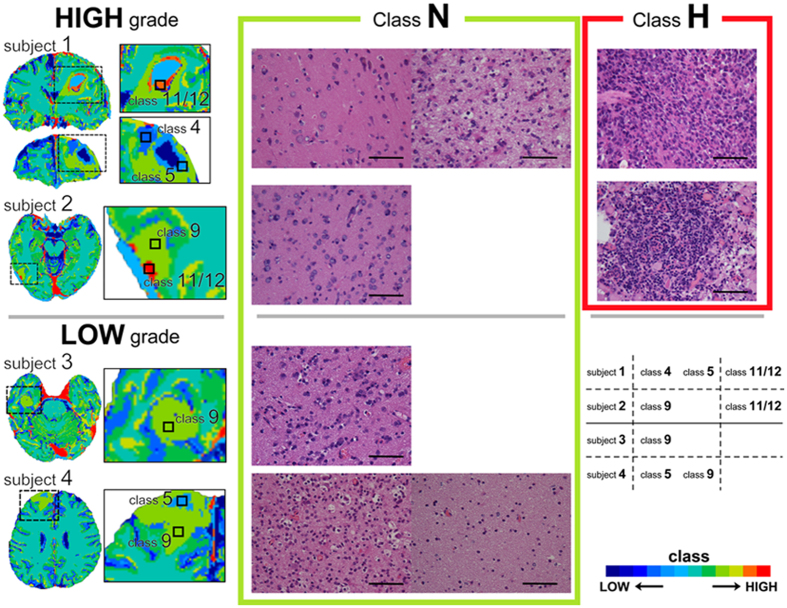
The 12-class MR-based clustered images and the pathological features of resected tissues (stained with hematoxylin and eosin) in each class for four patients, including classes 4 (Class N), 5 (Class N), 9 (Class N), 11 (Class H), and 12 (Class H). The pathological diagnoses of subjects 1 and 2 were high-grade gliomas and those of subjects 3 and 4 were low-grade gliomas, all of which were corresponded to the preoperative predictions by MR-based clustered images. The pathological hallmarks of high-grade gliomas are presented in Class H (right column), and the atypical features and hypercellularity found in HGG are not seen in Class N (middle column). Scale bars, 100 μm.

We also developed 12-class MRcIs in four new patients who were clinically suspected to be gliomas using the previous unsupervised clustered data obtained from the 36 patients. The number of clusters was tentatively chosen because 12-class MRcIs showed the best classification performance. MRcIs were automatically transferred to the Brainlab Neuronavigation interface (BrainLab AG, Feldkirchen, Germany), thereby providing real-time cross-sectional images of the brain intraoperatively. Using this interface, we obtained tissue sections in the classes 4 (Class N), 5 (Class N), 9 (Class N), 11 (Class H), and 12 (Class H). Two tissue samples for Class N and a sample for Class H in the subject 1, one for Class N and one for Class H in the subject 2, one for Class N in the subject 3, and two for Class N in the subject 4, respectively (Fig. 6). Therefore, six tissue samples for Class N and two for Class H were totally evaluated. Unfortunately, Class L tissue sections were not obtained because the correction of the voxels within the tumors was considerably small on the neuronavigational interface for these patients. In the Class H pathological tissue samples, we observed atypical tumor cells that were hypercellular with mitotic figures along with vascular proliferation characterized as HGG. Conversely, the classes 4, 5 and 9 which were defined as Class N showed that cellularity was moderately increased; however, no other anaplastic features such as necrosis, destruction, or neovascularization were observed (Fig. 6).

## Discussion

We investigated a two-step clustering approach using SOM followed by KM to visualize the grade of glioma with MRI and to distinguish HGGs from LGGs. MRcIs enabled us to predict the glioma grading even though they were calculated from preoperative images. Next, we assessed the validity of MRcIs for glioma grading in a supervised manner using SVM. The 12-class MRcIs exhibited the highest classification performance for predicting the glioma grade. The classifier in the 12-class MRcIs showed that the ratios of the classes 11 and 12 were significantly higher in HGGs and those of the classes 1 and 2 were significantly higher in LGGs. Four new patients underwent individual MRcIs for intraoperative pathological examination; the Class H tissues showed pathological findings of HGG, whereas the Class N tissues indicated LGG. Although the relationship between the class and pathology should be clarified by examining more patients, the findings of the MRcI-guided tissue sampling suggest that regional glioma grading is possible.

Although the method was largely based on that described by Inano *et al*.^17^, we improved some methodological points. First, we introduced bias field correction implemented as the “unified segmentation” algorithm of SPM8^21^,^22^ to reduce the effect of spatially varying homogeneity in MRcIs. Because bias field correction was not considered in our previous study, clustered images revealed spatially varying homogeneity like a color gradient, which sometimes makes it difficult to predict the boundary when the boundary class of tumor is the same as the outside of tumor. The bias field correction technique made clustered images easier to understand grading. Second, BraTumIA^23^ was newly introduced in this study to define regions of interest (ROIs) automatically. Using the software, anyone can reproducibly define the same ROI. It is important to define ROIs as masks for the effective bias field correction. Furthermore, the abnormal areas of BraTumIA were sometimes located in multiple parts of the brain except tumor, we need to check the number of tumors using conventional MRI to exclude the possibility of multifocal gliomas. If a new patient would have multifocal gliomas, we would need to select two or more ROIs as masks.

In most previously conducted studies, only one feature was used for grading^7^,^8^, and the results of those studies are still controversial. Although some of these studies used multiple features for grading in a supervised manner, they needed preoperatively to decide pathology of each area, such as edematous tissue, LGG, HGG, because a supervised manner needs labeled data. We previously reported a novel unsupervised method that does not need pathological information in advance. In that study, we found that multiple features from DTIs were useful for glioma grading^17^. We further used more conventional MRIs in the present study, and the multiple features of these conventional MRIs were also useful. Although the T1WIce was remarkable in Class H and was suspected to be a key feature for grading, some enhanced areas were not involved in Class H. When considering only T1WIce, both malignant and nonmalignant areas can be included and the grade of glioma cannot be determined. This may be the reason why past studies had different results for enhanced tumor regions. Furthermore, it is difficult for clinicians to determine where is enhanced especially on slightly enhanced regions. As to our dataset, the number of patients whose enhanced regions within tumor were automatically calculated from BraTumIA was 27 and the accuracy of preoperative tumor grading was 0.833. Using our clustering methods for only T1WIce as a single feature, the accuracy of pre-op tumor grading was 0.822, which was worse than our original result (0.863). Although using several MR images can facilitate tumor grading by allowing comparison of the images on the basis of the individual MRI characteristics, they are not always sufficient for clinicians because it is difficult to distinguish slight differences or relationships by the visual inspection of the images. Our method can form a clear clustered image summarized by merging the information from the different types of MRIs; each voxel contains information for tumor grading. Clinicians can easily grade the gliomas by the visual inspection of the clustered images. Thus, our novel method is advantageous because of multiple MRI features, particularly in clinical situations, and shows the potential of using multiple features.

The chart patterns of Class H comprised higher T1WIce values than the other Classes. In HGGs, the voxels of Class H without vessels were observed and were found to spread like a stain within a tumor. However, the enhanced regions were not always clustered in Class H, which were significantly higher in HGGs. This result suggests that T1WIce cannot sufficiently predict glioma grade, which may explain why previous studies could not show the role of contrast enhancement in tumor grading^6^,^7^,^8^. Therefore, we focused on contrast-enhanced areas within tumors to clarify the role of T1WIce. Class N was seen in both LGGs and HGGs, whereas Class H was mostly seen in HGGs, as shown in Fig. 3. This result may indicate that previous reports could not grade the gliomas as they only used enhancement information. Contrast enhancement is a sign of a leaky blood–brain barrier, and is related to the neovascularity of the tumor^6^. Conversely, some authors have reported that approximately 32–40% of nonenhancing glial tumors contain histologically anaplastic components^8^,^24^. This means that tumor enhancement may be due to the formation of capillaries with an inadequate blood–brain barrier, rather than due to the active destruction of the existing blood–brain barrier^25^. Another study also showed that there is a correlation between tumor vascular density and cell proliferation rate^26^. These studies may indicate that LGG can sometimes be enhanced. Conversely, regarding T2WI, Class L had high T2 values, whereas Class H did not. Edematous tissue is defined as tissue with a high T2WI signal intensity^27^, and T2WI hyperintensity is sometimes considered to indicate edematous tissue; however, the peritumoral hyperintensity on T2WI is nonspecific and represents tumor infiltration, vasogenic edema, or both^11^. Although the classes 3, 5, and 9 also had high T2WI values, they were included in Class N. This means that some areas showing T2WI hyperintensity are specific to LGGs, whereas others are not. Further, FLAIR can be used to assess abnormalities in the white matter; however, hyperintensity on FLAIR can indicate edema and/or tumor cell infiltration. We used two-dimensional T2WI and FLAIR data and coregistered them with T1WI. The accuracy of the results may increase if three-dimensional T2WI and FLAIR images are applied. However, when considering their clinical application, three-dimensional T2WI and FLAIR are uncommon, particularly in local hospitals, and our methods may be sufficiently useful for predicting preoperative tumor grading.

The 12-class MRcIs had the best classification performance, and the numbers may be justified when considering MRI for brain tumors because the following can be identified using this imaging modality: white matter, gray matter, cerebrospinal fluid, high-grade tumors, low-grade tumors, infiltration of tumor cells, edema, necrosis, gliosis, hemorrhage, cystic lesions, fibrous change, and calcification. However, our goal was not to distinguish between these tissues, but to develop a method of determining the parts of brain tumors that are the most likely to be malignant. We believe that our additional categorization of the three “Classes” allows improved understanding of each class. We defined the classes that were significantly higher in HGGs as “Class H,” those that were significantly higher in LGGs as “Class L,” and those that did not contribute to the grading decision as “Class N.” This method of categorization was effective from two points of view. First, the suspected malignant areas can be visualized; thus, we do not need to further confirm the pathology of the tissue. Second, organizing more voxels can make the resection of the tumor via neuronavigational guidance easier because the voxel numbers for some classes of MRcIs were considerably small to be visualized on the neuronavigational system and to be resected as a separate class. It should be noted that the categorization of the three groups differs from the original smaller number of classes such as four because we categorized it according to the results of the 12-class MRcIs, which showed the best classification performance. In addition, the patterns of the values of the classes 11 and 12 in 12-class MRcIs were similar to those of the 8- to 16-class MRcIs. The patterns of the values of the classes 1 and 2 in the 12-class MRcIs were also similar to those of the 10- to 20-class MRcIs. Although the 12-class MRcIs showed the best performance, the other classes of clusters, except for the 4-class MRcIs, also showed high AUCs, with values over 0.85. These results suggest that our method is robust for classification regardless of the number of clusters, particularly for those over six.

We successfully used our approach to visualize the grade and heterogeneity of gliomas; furthermore, we prospectively applied our method to four new patients in order to confirm the utility of our proposal method and the possibility of regional glioma grading. The pathological features were atypical tumor cells that were hypercellular with mitotic figures along with vascular proliferation, thereby suggesting a suspected malignant tumor in the pathological tissue samples of Class H. Moreover, although cellularity was moderately increased, no other anaplastic features such as necrosis, destruction, or neovascularization were identified in the tissue samples of Class N (Fig. 6). The overall accuracy of the two tissues for Class H was 100% (2/2) according to their histological grades. Furthermore, the pathological features of all the six tissue samples for Class N did not show any anaplastic features. These results suggest that regional grading prediction of Class H using MRcIs is consistent with pathological results. Although the excellent diagnostic accuracy of machine tissue classification for eight tissue samples in the four patients, the sample size remains small for a clinically meaningful conclusion and further studies are needed with more patients, particularly in terms of validating the diagnostic accuracy of machine tissue classification. Because of the heterogeneity of gliomas, diagnosis by biopsy is sometimes inaccurate^5^; the high-spatial resolution of T1WI, T1WIce, T2WI, and FLAIR may be better for the correct resection of the suspected malignant area to circumvent the limitation of our previous study involving the use of DTI, which has a low-spatial resolution. Although we showed only the 12-class MRcIs showing the best performance as representative cases and applied to new patients, all the AUCs among the 6-, 8-, 10-, 12-, 16-, and 20-class MRcIs consistently revealed good classification performances over 0.86 shown in Fig. 4A (left). Furthermore, shown in Supplementary Fig. S1, the chart patterns of Classes H and L in all numbers of K except 4-class demonstrated a similar pattern to those in the 12-class MRcIs. The distribution patterns of classes H and L in MRcIs of all numbers of K except 4 were also similar. Although we tentatively chose the 12-class MRcIs showing the best performance in order to obtain pathological tissues in each class of an MRcI for new tumors, the distribution patterns of classes H and L regardless of numbers of K except 4 were similar in this study. However, a future study with more patients will be needed to validate the optimal number of clusters. Although further studies are needed with more patients, our results suggest the potential of regional glioma grading using MRcIs. Further studies may be able to clarify the difference between “malignant” and “benign” enhanced areas in patients, and can help in surgical strategy, such as identifying the tumor regions that must be resected for good prognosis. Unfortunately, non-enhancing tumor regions characterized as Class H were not found in the new four patients or no apparently non-enhancing tumor characterized as Class H was found in the training cohort. It would be important to show apparently non-enhancing tumor characterized as Class H and confirmed in histology as high grade. In addition to pathological features, genetics subtypes such as IDH1/2 mutations would be important. As our study was retrospective study, as to the IDH1 mutation, only 13 out of 21 HGG patients were examined. Two of 10 grade IV gliomas who were examined the sequence of the IDH1 gene revealed IDH1 mutation and eight of 10 did not reveal IDH1 mutation. Two of three grade III gliomas who were examined the sequence of the IDH1 gene revealed IDH1 mutations and one of three did not reveal the IDH1 mutation. The survival outcomes were as the following: all two grade IV patients with IDH1 mutation lives at least 32 months, whereas one out of two grade III patient without IDH1 mutation only survived 9 months. Mutation of IDH1 gene would be associated with improved outcome^28^ even in our small dataset. The genetic subtypes are one of the prognostic factors and can be used as supervised learning adding to the pathological features in prospective study. Applying our method, we may be able to predict the genetic subtypes by preoperative images in the future. More extensive surgical resection of gliomas is associated with longer life expectancy^1^; therefore, if tumors can be removed with minimal volume of resection, the risk of functional deficit may be decreased, which can improve the quality of life for patients.

## Materials and Methods

The overview of the study procedure of the present study is depicted in Fig. 1 and is summarized below:

Clustering stage:

1. Intensity normalization of the bias field-corrected MR images.
2. Feature extraction from intensity-normalized T1WI, T1WIce, T2WI, and FLAIR images.
3. Clustering of the input vectors from the feature extraction using SOM followed by KM.
4. Visualization of the whole brain in MRcIs. Classification stage:
5. Calculation of class ratios by MRcIs within the regions of interest (ROIs).
6. Classification using MRcIs by SVM.

We also applied our method prospectively to four new patients in order to confirm the utility of our proposal method and the possibility of regional glioma grading. The additional overview of the prospective study procedure is summarized below:

MRcI-guided tissue sampling stage:

1. Construction of MRcIs for new patients according to the clustering stage.
2. Resection of tissue from representative classes in MRcIs during operation.
3. Comparison of pathological tissue samples with the corresponding MRcIs.

### Subjects

We retrospectively reviewed 36 patients (23 men, 13 women) with gliomas who underwent DTI, T1WI, T1WIce, T2WI, and FLAIR sequences in our study^17^. 32 out of 33 patients in the previous study were also involved though we did not use the same images, that is, DTIs. One patient whose MR images except DTI were obtained using another MRI machine was excluded and four more patients whose MR images were obtained using the same MRI were added in this study. They were aged between 16 and 83 years and had newly diagnosed and histologically confirmed diffusely infiltrative gliomas as defined by the WHO classification^29^ at the Kyoto University Hospital between March 2010 and June 2013. Tumor resections were performed on 35 patients, and a biopsy was performed on one patient who has gotten the diagnosis as anaplastic oligoastrocytoma, WHO grade III, classified as HGG. Twenty-one tumors were located in the frontal region, nine in the temporal, three in the parietal, one in the occipital, and two in the frontoparietal (Table 1). The present study was approved by the Ethics Committee of the Kyoto University Graduate School of Medicine (C 570) and written informed consent was obtained from all patients. This study was conducted in accordance with the Declaration of Helsinki. Additional details of the MRI acquisition of the study are provided in Supplemental Information.

### MRI post-processing

The MRI data were analyzed using SPM8 (Statistical Parametric Mapping software; Wellcome Trust Centre for Neuroimaging, London, UK; http://www.fil.ion.ucl.ac.uk/spm). First, T1WIce, T2WI, and FLAIR were coregistered with each T1WI and resliced with the 4th degree B-spline interpolation to match with T1WI voxel-for-voxel^21^ using SPM8. The data were corrected for bias field signals, which corrupt MR images spatially^22^, with the “unified segmentation” algorithm of SPM8 using the default settings (warp regularization = 1, warp frequency cut-off = 25, bias regularization = 0.0001, bias FWHM = 60, and voxel size = 1 mm × 1 mm × 1 mm)^21^,^30^,^31^.

The present study was largely based on the method of voxel-based DTI clustering described by Inano *et al*.^17^ with some modifications. First, the features for unsupervised clustering were extracted from the voxels on the four MR sequences, namely T1WI, T1WIce, T2WI, and FLAIR, with samples at every 125 (5 × 5 × 5) voxels within the binary whole brain mask image calculated by gray matter, white matter, and cerebrospinal fluid images, which were obtained based on T1WI with the “unified segmentation” algorithm of SPM8 (Segment)^21^. The number of extracted features was 161,157 ± 1853 (mean ± standard deviation) for each subject. The features of all subjects were stacked and used for the input vectors. The components of each input vector were extracted from T1WI, T1WIce, T2WI, and FLAIR images in this study. Second, the extracted feature vectors were used for calculating voxel-based clustered images. We applied a two-level clustering approach using a batch-learning self-organizing map (BLSOM)^15^ and the K-means++ (KM++) algorithm^32^ for unsupervised clustering. The results from BLSOM are more consistent than those of the standard sequential SOM due to the independence of the input order^33^, and the KM++ algorithm improves both the speed and accuracy compared with the classic KM algorithm^32^. Following our previous study, we chose the numbers of *K* = 4, 6, 8, 10, 12, 16, 20. We implemented this two-level clustering algorithm using the in-house SOM software, which enabled the use of BLSOM followed by KM++. Third, after unsupervised clustering by BLSOM followed by KM++, 400 protoclusters (weighted vectors) with *K*-class label information were generated. The label information of the nearest protocluster was assigned to each voxel on the four intensity-normalized MR images within the binary whole brain mask image. Then, voxel-based images with *K*-class label information were obtained, namely the MRcIs (Fig. 1). The common logarithmic value of the ratio of each class in MRcIs was calculated from ROIs defined by BraTumIA described in the next section in each subject and used to input the features to the SVM^34^.

### Definition of ROIs

We calculated ROIs from the abnormal areas of the BraTumIA^23^. ROIs from BraTumIA were used for the following two purposes: for the “Masking image” of the “Segment” in SPM and for the calculation of the common logarithmic value of the ratio of each class in MRcI in each patient. ROIs included necrotic tissue, active enhancing tumor tissue, nonenhancing tumor tissue, and edematous tissue. The number of voxels in each ROI ranged from 2541 to 306,000. Because the abnormal areas of BraTumIA were sometimes located in different parts of the brain, we checked the main part of the tumor using by T1WI, T1CE, T2WI and FLAIR and manually defined only one ROI, because all patients had only one tumor mass in the study. There was no other manual editing in removing ROIs during incomplete skull stripping. The co-registration function in SPM was used instead of the built-in co-registration from BraTumIA. We used these ROIs for the “Segment” of SPM as a tumor mask set as the “Masking image”, which forces the masked area not to contribute when estimating parameters, in the previous subsection and for SVM, as described in the next section. Note that ROIs were not used for unsupervised clustering or for creating MRcIs.

### Classification using MRcI: SVM

A linear kernel SVM^34^ was chosen as a classifier to distinguish between LGGs and HGGs and the hyperparameter (C) of the linear kernel SVM was optimized according to the method described in a practical guide to SVM classification^35^ and in our previous study^17^.

A LOOCV strategy was also used to assess the classification performance that is widely used in machine learning and allows the use of most of the data for training^36^. The decision function derived from the training datasets is used to classify or calculate a decision value for the test subject. After all the 100 times LOOCV were repeated, the mean accuracy, sensitivity, and specificity for all the folds are calculated, respectively. We also evaluated the decision values^37^ for receiver operating characteristic (ROC) curves and AUC, and the CIs of these estimates calculated using 100 times LOOCV. We used C++ and the LIBSVM library (Chang and Lin, 2011; software available at http://www.csie.ntu.edu.tw/~cjlin/libsvm) to implement a linear kernel SVM with a two-step grid search technique for hyperparameter (C) and a LOOCV strategy.

### Statistical analysis

Statistical analysis was performed in accordance with our previous study^17^. We determined if the classification performances significantly different according to K in the KM++ method (K = 4, 6, 8, 10, 12, 16, 20), and analyzed the AUCs for the different numbers of K by one-way analysis of variance followed by Tukey’s multiple comparison tests. Differences were considered to be statistically significant when p < 0.05.

The behavior of the classifier in the K class with the best classification performance was evaluated using the pROC library in R to generate ROC curves with 95% CIs computed with 2000 stratified bootstrap replicates^17^.

Wilcoxon–Mann–Whitney tests with exact p values and CIs calculated by a permutation test followed by a Bonferroni correction for multiple comparisons were used to compare the log-ratio values of each class in the K class with the best classification performance between LGG and HGG groups^38^. The differences between the groups were considered to be statistically significant when p < 0.05/K.

The ratios of the normalized intensities on the four MR images of each class in the *K* class with the best classification performance were analyzed with bootstrapped 95% CIs. Then, we used bootstrapped 95% CIs to analyze the ratios of the normalized intensities on three of the MR sequences, namely T1WI, FLAIR, and T2WI, within the enhanced tumor regions when *K* revealed the best classification performance. The statistical software package R, version 3.0.2 (The R Foundation for Statistical Computing, http://www.r-project.org/), was used to perform all statistical analyses.

## Additional Information

**How to cite this article**: Inano, R. *et al*. Visualization of heterogeneity and regional grading of gliomas by multiple features using magnetic resonance-based clustered images. *Sci. Rep.* **6**, 30344; doi: 10.1038/srep30344 (2016).

## Supplementary Material

Supplementary Information

## Figures and Tables

**Table 1:** Summary of patient data.

Histopathology	n	WHO grade	Age (years)	Location
**high-grade gliomas**	**21**	**III and IV**	**53.7 ± 17.7**	
glioblastoma	15	IV	56.6 ± 16.3	frontal, parietal, temporal, frontoparietal
anaplastic astrocytoma	4	III	33.5 ± 4.8	frontal, temporal
anaplastic oligoastrocytoma	2	III	72.0 ± 5.0	frontal
**low-grade gliomas**	**15**	**II**	**43.5±13.3**	
diffuse astrocytoma	7	II	48.6 ± 14.9	frontal, parietal, frontoparietal
oligoastrocytoma	3	II	40.3 ± 15.5	frontal, temporal
oligodendroglioma	4	II	38.0 ± 3.7	frontal, temporal
mixed oligoastrocytoma	1	II	40.0	frontoparietal

Age (years) is given as means ± standard deviation.
