# Supplementary material for: Visualization of heterogeneity and regional grading of gliomas by multiple features using magnetic resonance-based clustered images
Source: Sci Rep. 2016 Jul 26;6:30344. doi: 10.1038/srep30344 (PMC4960553; doi:10.1038/srep30344)
Supplement: Supplementary Information [file srep30344-s1.doc]

**Supplemental Information**

**Visualization of heterogeneity and regional grading of gliomas by multiple features using magnetic resonance-based clustered images.**

Rika Inano^a,b^ Naoya Oishi^b,c^, Takeharu Kunieda^a^, Yoshiki Arakawa^a^, Takayuki Kikuchi^a^, Hidenao Fukuyama^b,d^, Susumu Miyamoto^a^

^a^Department of Neurosurgery, Kyoto University Graduate School of Medicine, Kyoto, Japan

^b^Human Brain Research Center, Kyoto University Graduate School of Medicine, Kyoto, Japan

^c^Department of Psychiatry, Kyoto University Graduate School of Medicine, Kyoto, Japan

^d^Center for the Promotion of Interdisciplinary Education and Research, Kyoto University, Kyoto, Japan





**Supplementary Figure 1.** Strip chart and box plots showing the median, interquartile range, inner fence, and outliers (circles) for log-ratio values of each class MR-based clustered images of low-grade (light blue) and high-grade (red) gliomas. ***p* < 0.001, **p* < 0.005 (Class L < Class H), ^†^*p* < 0.005, ^††^*p* < 0.001 (Class H < Class L), exact Wilcoxon–Mann–Whitney tests (left). The plots show the AUC versus the numbers of *K* in the KM method. The values are presented as means and error bars, with 95% CIs (light blue-shaded area).

**
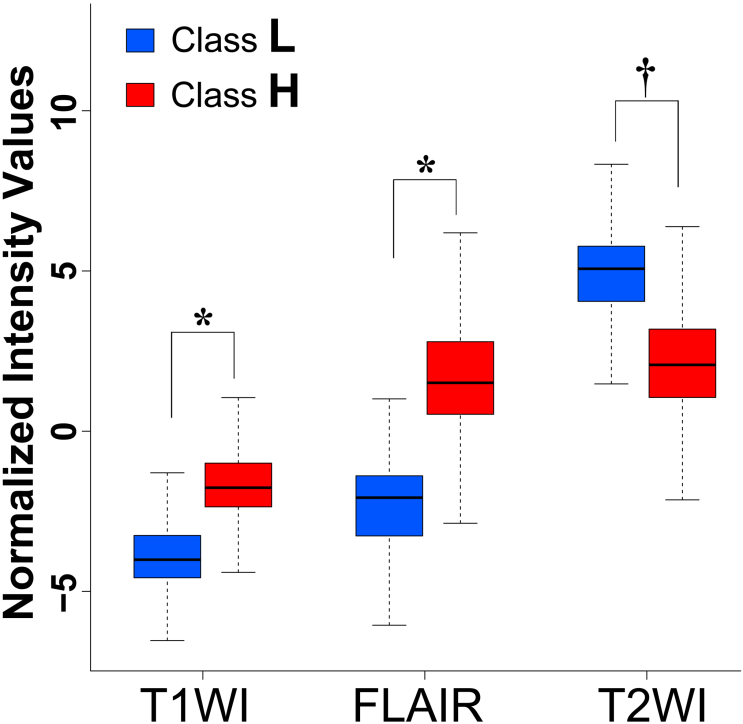
**

**Supplementary Figure 2.** Strip chart showing the median, interquartile range, and inner fence for normalized intensity values of each MRI in Class L (blue) and Class H (red) within the enhanced tumor regions. **p* < 10^-16^ (Class L < Class H), ^†^*p* < 10^-16^ (Class H < Class L), exact Wilcoxon–Mann–Whitney tests.

**MRI data acquisition**

MR images were obtained using a 3-Tesla Trio Tim (Siemens, Erlangen, Germany) equipped with a 32-channel phased array head coil. MP-RAGE was used to acquire three-dimensional T1WI with the following parameters: repetition time (TR) = 1900 ms, echo time (TE) = 2.58 ms, inversion time (TI) = 900 ms, flip angle = 9°, field of view (FOV) = 230 × 230 mm, slices = 256, and voxel size = 0.9 × 0.9 × 0.9 mm. The parameters of the T1 VIBE fat-saturated images were: TR = 6 ms, TE = 2.26 ms, flip angle = 15°, FOV = 230 × 230 mm, slices = 256, and voxel size = 0.9 × 0.9 × 0.9 mm. The parameters of T1WIce were: TR = 1900 ms, TE = 2.58 ms, flip angle = 9°, FOV = 230 × 230 mm, slices = 256, and voxel size = 0.9 × 0.9 × 0.9 mm. The parameters of T2WI were: TR = 3200 ms, TE = 79 ms, flip angle = 120°, field of view = 224 × 150 mm, slices = 35, and slice thickness = 3.0 mm. The parameters of FLAIR were: TR = 12,000 ms, TE = 100 ms, TI = 2760 ms, flip angle = 120°, FOV = 224 × 182 mm, slices = 35, and slice thickness = 3.0 mm. T1WIce were obtained using gadolinium (0.2ml/kg) without dynamic enhancement.
